# Prognostic and Therapeutic Value of Apolipoprotein A and a New Risk Scoring System Based on Apolipoprotein A and Adenosine Deaminase in Chronic Lymphocytic Leukemia

**DOI:** 10.3389/fonc.2021.698572

**Published:** 2021-07-01

**Authors:** Xiaoya Yun, Xiang Sun, Xinting Hu, Huimin Zhang, Zixun Yin, Xin Zhang, Ming Liu, Ya Zhang, Xin Wang

**Affiliations:** ^1^ Department of Hematology, Shandong Provincial Hospital, Cheeloo College of Medicine, Shandong University, Jinan, China; ^2^ Department of Hematology, Shandong Provincial Hospital Affiliated to Shandong First Medical University, Jinan, China; ^3^ School of Medicine, Shandong University, Jinan, China; ^4^ Shandong Provincial Engineering Research Center of Lymphoma, Jinan, China; ^5^ Branch of National Clinical Research Center for Hematologic Diseases, Jinan, China; ^6^ National Clinical Research Center for Hematologic Diseases, The First Affiliated Hospital of Soochow University, Suzhou, China

**Keywords:** chronic lymphocytic leukemia (CLL), apolipoprotein A, prognosis, L-4F, lipid metabolism

## Abstract

Lipid metabolism is related to lymphomagenesis, and is a novel therapeutic target in some hematologic tumors. Apolipoprotein A (ApoA), the major protein of high-density lipoprotein (HDL), plays a crucial role in lipid transportation and protecting against cardiovascular disease, and takes effect on anti-inflammation and anti-oxidation. It is correlated with the prognosis of some solid tumors. Yet, there is no investigation involving the role of ApoA plays in chronic lymphocytic leukemia (CLL). Our retrospective study focuses on the prognostic value of ApoA in CLL and its therapeutic potential for CLL patients. Herein, ApoA is a favorable independent prognostic factor for both overall survival (OS) and progression-free survival (PFS) of CLL patients. ApoA is negatively associated with β2-microglobulin (β2-MG) and advanced stage, which are poor prognostic factors in CLL. Age, Rai stage, ApoA, and adenosine deaminase (ADA) are included in a new risk scoring system named *ARAA-score*. It is capable of assessing OS and PFS of CLL patients. Furthermore, cell proliferation assays show that the ApoA-I mimetic L-4F can inhibit the proliferation of CLL cell lines and primary cells. In conclusion, ApoA is of prognostic value in CLL, and is a potential therapy for CLL patients. The *ARAA-score* may optimize the risk stratification of CLL patients.

## Introduction

Chronic lymphocytic leukemia (CLL) is a heterogeneous disease characterized by the proliferation of small and mature malignant B-lymphocytes co-expressing CD5 and CD23 (1). Although novel therapies improve the survival of CLL patients, such as the Bruton tyrosine kinase (BTK) inhibitor ibrutinib and the B-cell lymphoma 2 (Bcl2) inhibitor venetoclax, high-risk CLL remains incurable (2–5). In the last 40 years, a series of prognostic biomarkers have been identified to evaluate the prognosis. The high levels of lymphocyte doubling time (LDT), serum beta2-microglobulin (s-β2-MG), serum thymidine kinase (s-TK), and lactic dehydrogenase (LDH) are associated with poor survival of CLL patients. CD38 positive and ZAP70 positive both show advanced progression. Unmutated IGHV status is an adverse prognostic marker for CLL patients. Del17p/p53 mutation and CD49d positive have both prognostic and predictive value in CLL (6–10).

Lipid metabolism is related to lymphomagenesis and is a novel therapeutic target for hematologic tumors (11–13). CLL cells are like adipocytes rather than normal B-lymphocytes or other leukemia cells, producing energy by utilizing free fatty acids (FFA) (14). The overexpression of some proteins involved in lipid metabolism was found in CLL cells (15). Statins, the competitive inhibitors of 3-hydroxy-3-methyl glutaryl coenzyme A (HMG-CoA) reductase, induce apoptosis in CLL cells (16). Otherwise, statins use can reduce CLL risk with a dose-response effect (17). Ibrutinib, the first choice for CLL patients, inhibits the FFA metabolism *in vitro* and disrupts energy production (18). On the other hand, it increases the level of high-density lipoprotein (HDL) in CLL patients, whereas a pronounced drop was found in HDL level of CLL patients 3 to 4 years before the diagnosis (12, 19). The lipid-related pathway may be a potential target for novel CLL therapies.

Apolipoprotein A (ApoA), the main component of HDL, is known as a favorable lipoprotein and plays a role in reverse cholesterol transport and protecting against cardiovascular disease. ApoA-I, the major subtype of ApoA, is mainly produced by hepatocytes and catabolized in the liver (20). Besides regulating cholesterol trafficking, it participates in the innate humoral immune and has anti-inflammation potential, as well as anti-oxidation ability (21). ApoA-I mimetics, comprising 18 amino acids, mimic the functions of ApoA-I and are used to treat atherosclerosis (22).

Herein, we conduct a retrospective study for the first time to investigate the effect that ApoA takes on the prognosis and therapy of CLL patients. Furthermore, we establish a new risk scoring system to optimize the risk stratification of CLL patients. The study also investigates the association between ApoA and other clinical indicators of CLL patients. Altogether, our study may improve the risk stratification of CLL patients and provide a potential therapeutic target for them.

## Materials and Methods

### Patients

Our study included 150 CLL patients who were newly diagnosed in Shandong Provincial Hospital Affiliated to Shandong University from January 2010 to December 2019. All patients met the revised International Workshop on Chronic Lymphocyte Leukemia (IWCLL) diagnostic criteria. The definitions of the overall survival (OS) time and the progression-free survival (PFS) time were also based on it (23). The last follow-up was performed in April 2020. The study was ratified by the Hospital Ethics Committee in Shandong Provincial Hospital Affiliated to Shandong University in China. All participants signed informed consents. All data were performed following the Declaration of Helsinki.

### Cell Lines and Regimens

The human p53 deleted/mutated CLL cell line MEC1 cells and the human p53 wide-type CLL cell line EHEB cells were obtained from Professor Liguang Chen from Moores Cancer Center, University of California, San Diego and American type culture collection (ATCC), separately. Primary cells were extracted from peripheral blood or bone marrow from 4 CLL patients newly diagnosed in Shandong Provincial Hospital affiliated to Shandong University from September to October 2020. MEC1 cells were suspended in IMDM medium while EHEB cells and primary cells were maintained in RPMI-1640 medium, with 10% FBS (Gibco, MD, USA), 1% penicillin/streptomycin, and 2 mM l-glutamine. Cells were incubated at 37°C in 5% CO_2_. L-4F (Ac-D-W-F-K-A-F-Y-D-K-V-A-E-K-F-K-E-A-F-NH2, synthesized by GL Biochem Ltd, Shanghai, China) was dissolved in water to 1 mg/ml and freshly prepared for each use (24).

### Cell Proliferation Assay

The proliferation assay of CLL cells was performed with the Cell Counting Kit-8 (CCK-8; Dojindo, Kumamoto, Japan) as described. Three replicates were performed for each sample (25–27). MEC1, EHEB, and primary cells were treated with L-4F at the concentration between 10 and 250 µg/ml for 48 h. After that, CCK-8 (10 µM) was added to the cells for an additional 3 h. The absorbance was measured at 450 nm.

### Statistical Analysis

SPSS 26.0 and Graphpad Prism 5.0 were used to analyze the data. The Kaplan-Meier curves were used to assess survival rates, while the log-rank tests were used for comparison. Cox proportional hazard regression model was used to verify prognostic independence. Pearson and Spearman tests were performed to evaluate the correlation between ApoA and other serum parameters. Chi-square tests were used to assess the correlation between ApoA and clinical stage, cytogenetic aberrations, and IGHV mutation status. ROC curves were used to compare the Binet stage and the new risk scoring system we developed. *P*-values < 0.05 were considered statistically significant.

## Results

### Univariate and Multivariate Cox Proportion Hazard Regression Analysis of OS of CLL Patients

**Figure 1:**
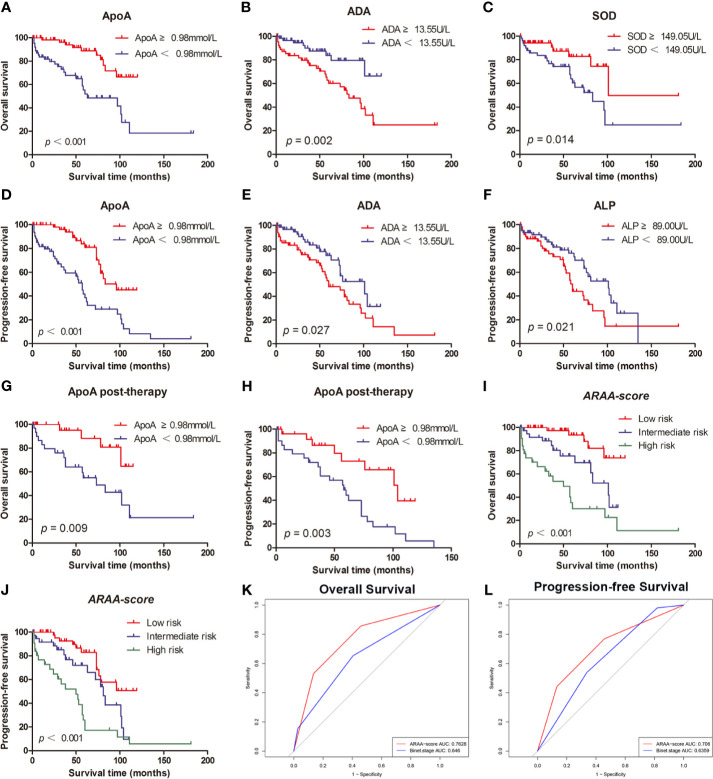
**(A)** The high level of ApoA is associated with better OS of CLL patients. **(B)** The high level of ADA is associated with better OS of CLL patients. **(C)** The high level of SOD is associated with poor OS of CLL patients. **(D)** The high level of ApoA is associated with better PFS of CLL patients. **(E)** The high level of ADA is associated with poor PFS of CLL patients. **(F)** The high level of ALP is associated with poor PFS of CLL patients. **(G)** The high level of ApoA post-therapy is associated with better OS of CLL patients. **(H)** The high level of ApoA post-therapy is associated with better PFS of CLL patients. **(I)** *ARAA-score* is associated with OS of CLL patients. **(J)** *ARAA-score* is associated with PFS of CLL patients. **(K)** *ARAA-score* is superior to Binet stage for OS of CLL patients. **(L)** *ARAA-score* is superior to Binet stage for PFS of CLL patients.

The associations between OS and parameters of lipid metabolism were analyzed. The clinical and laboratory data of all patients with CLL were shown in **Table 1**. Despite the known prognostic factors, including the age, Rai stage, Binet stage, and β2-MG, the Kaplan-Meier curves revealed markedly increased survival in patients with high ApoA level (*χ* ^2^ = 14.985; *p*<0.001; shown in **Figure 1A**), with low adenosine deaminase (ADA) level (*χ* ^2^ = 8.294; *p*=0.002; shown in **Figure 1B**) and with low superoxide dismutase (SOD) level (*χ* ^2^ = 6.030; *p*=0.014; shown in **Figure 1C**). The median OS time of patients with low ApoA level was 63 months, while that of patients with high ApoA level was not reached. The Cox proportion hazard model of OS of CLL patients was shown in **Table 2**. In univariate analysis, age (*p*=0.010), Rai stage (*p*=0.002), Binet stage (*p*=0.006), β2-MG (*p*=0.014), ApoA (*p*<0.001), SOD (*p*=0.018), and ADA (*p*=0.006) were dramatically associated with OS. Then the prognostic factors significantly related to OS in univariate analysis were included in the multivariate Cox proportion hazard regression analysis. The multivariate analysis showed that age (*p*=0.026), Rai stage (*p*=0.025), and ApoA (*p*=0.028) were independent prognostic factors of CLL patients.

**Table 1:** Clinical and laboratory features of chronic lymphocytic leukemia patients.

Parameters	N	Mean ± SD	Range	Medium
**Age, years**	150	61.35 ± 9.89	36–83	62
**LDH, U/L**	126	239.78 ± 194.23	80.00–1874.00	193.75
**β2-MG, mg/L**	142	3.50 ± 1.83	1.28–14.91	3.05
**TG, mmol/L**	150	1.28 ± 0.70	0.41–4.13	1.10
**TC, mmol/L**	150	4.26 ± 1.17	1.07–9.89	4.07
**HDL-C, mmol/L**	150	1.03 ± 0.32	0.15–2.30	0.98
**LDL-C, mmol/L**	150	2.69 ± 0.87	0.86–6.86	2.57
**sdLDL-C, mmol/L**	43	0.93 ± 0.43	0.29–2.59	0.84
**ApoA, g/L**	150	0.99 ± 0.25	0.11–1.92	0.98
**ApoB, g/L**	150	0.89 ± 0.30	0.37–2.14	0.86
**ApoA/ApoB**	150	1.20 ± 0.45	0.29–2.94	1.14
**HCY, µmol/L**	102	12.85 ± 4.52	5.07–26.44	12.12
**Lipo a, g/L**	148	0.22 ± 0.21	0.00–1.00	0.16
**Lp-PLA2, IU/L**	24	635.50 ± 266.90	143.00–1379.00	522.50
**FFA, mmol/L**	79	0.52 ± 0.31	0.05–2.20	0.49
**AST, U/L**	147	23.99 ± 15.84	6.00–127.00	20.00
**ALT, U/L**	147	20.05 ± 18.22	4.00–153.00	16.00
**SOD, U/L**	122	147.43 ± 30.45	81.00–274.00	149.05
**GGT, U/L**	147	30.20 ± 38.99	9.00–317.00	19.00
**ALP, U/L**	147	96.91 ± 43.61	34.00–346.00	89.00
**GLDH, U/L**	130	3.51 ± 3.38	0.50–21.40	2.55
**ADA, U/L**	146	14.88 ± 8.35	4.90–84.50	13.55
**TBA, µmol/L**	145	6.43 ± 6.90	0.30–60.20	4.50
**TBIL, µmol/L**	147	16.15 ± 12.89	5.30–114.90	13.00
**DBIL, µmol/L**	147	3.16 ± 2.49	0.90–16.30	2.40
**IBIL, µmol/L**	147	12.99 ± 10.80	4.40–98.60	10.60

LDH, lactate dehydrogenase; β2-MG, β2-microglobulin; TG, triglyceride; TC, total cholesterol; HDL-C, high-density lipoprotein-cholesterol; LDL-C, low-density lipoprotein-cholesterol; sdLDL-C, small dense low-density lipoprotein; ApoA, apolipoprotein A; ApoB, apolipoprotein B; HYC, homocysteine; Lipo a, lipoprotein a; Lp-PLA2, lipoprotein phospholipase A2; FFA, free fatty acid; AST, glutamic-oxalacetic transaminase; ALT, glutamic-pyruvic transaminase; SOD, superoxide dismutase; GGT, glutamyltranspeptidase; ALP, alkaline phosphatase; GLDH, glutamate dehydrogenase; ADA, adenosine deaminase; TBA, total bile acid; TBIL, total bilirubin; DBIL, direct bilirubin; IBIL, indirect bilirubin.

**Table 2:** Univariate and multivariate Cox proportion hazard model of potential prognostic parameters for overall survival of chronic lymphocytic leukemia patients.

Parameters		Univariate analysis			Multivariate analysis			
		*P*-value	HR	95%CI	*P*-value	HR	95%CI
**Age, years**	≥65 vs <65	**0.010**	**0.421**	**0.218**–**0.810**	**0.026**	**2.384**	**1.108**–**5.131**
**Sex**	male vs female	0.519	1.121	0.793–1.584			
**Rai stage**	III,IV vs 0,I,II	**0.002**	**0.342**	**0.172**–**0.681**	**0.025**	**2.788**	**1.137**–**6.833**
**Binet stage**	C vs A,B	**0.006**	**0.397**	**0.206**–**0.767**	0.662		
**LDH, U/L**	≥193.75 vs <193.75	0.095	1.815	0.901–3.655			
**β2-MG, mg/L**	≥3.50 vs <3.50	**0.014**	**2.368**	**1.194**–**4.696**	0.874		
**Del13q**	positive vs negative	0.135	2.007	0.805–5.007			
**Del11q**	positive vs negative	0.666	0.785	0.261–2.358			
**Del17p**	positive vs negative	0.602	0.744	0.245–2.262			
**Tri12**	positive vs negative	0.539	0.679	0.197–2.336			
**TG, mmol/L**	≥1.10 vs <1.10	0.965	0.985	0.517–1.879			
**TC, mmol/L**	≥4.07 vs <4.07	0.212	0.658	0.341–1.269			
**HDL-C, mmol/L**	≥0.98 vs <0.98	0.063	0.530	0.271–1.034			
**LDL-C, mmol/L**	≥2.57 vs <2.57	0.686	0.875	0.459–1.669			
**sdLDL-C, mmol/L**	≥0.84 vs <0.84	0.975	0.976	0.218–4.373			
**ApoA, g/L**	≥0.98 vs <0.98	**0.000**	**0.262**	**0.127**–**0.544**	**0.028**	**2.660**	**1.109**–**6.380**
**ApoB, g/L**	≥0.86 vs <0.86	0.060	0.522	0.265–1.027			
**ApoA/ApoB**	≥1.15 vs <1.15	0.065	0.533	0.274–1.040			
**HCY, µmol/L**	≥12.40 vs <12.40	0.995	1.003	0.421–2.388			
**Lipo a, g/L**	≥0.16 vs <0.16	0.980	0.992	0.519–1.897			
**Lp-PLA2, IU/L**	≥522.50 vs <522.50	0.249	0.214	0.016–2.943			
**FFA, mmol/L**	≥0.49 vs <0.49	0.181	0.475	0.160–1.414			
**AST, U/L**	≥20.00 vs <20.00	0.242	0.677	0.352–1.301			
**ALT, U/L**	≥16.00 vs <16.00	0.092	0.562	0.288–1.099			
**SOD, U/L**	≥149.05 vs <149.05	**0.018**	**0.375**	**0.165**–**0.848**	0.069		
**GGT, U/L**	≥19.00 vs <19.00	0.126	1.660	0.868–3.175			
**ALP, U/L**	≥89.00 vs <89.00	0.175	1.572	0.817–3.023			
**GLDH, U/L**	≥2.55 vs <2.55	0.507	0.794	0.403–1.567			
**ADA, U/L**	≥13.55 vs <13.55	**0.006**	**2.873**	**1.353**–**6.099**	0.050	0.431	0.185–1.000
**TBA, µmol/L**	≥4.50 vs <4.50	0.860	1.061	0.551–2.043			
**TBIL, µmol/L**	≥13.00 vs <13.00	0.411	1.315	0.684–2.528			
**DBIL, µmol/L**	≥2.40 vs <2.40	0.138	1.669	0.849–3.281			
**IBIL, µmol/L**	≥10.60 vs <10.60	0.373	1.346	0.700–2.586			

The bolded font means the parameters have statistical significances.

### Univariate and Multivariate Cox Proportion Hazard Regression Analysis of PFS of CLL Patients

The Kaplan-Meier curves revealed that there was a significant association between PFS and Rai stage (*χ* ^2^ = 6.820; *p*=0.009), Binet stage (*χ* ^2^ = 6.654; *p*=0.010), LDH (*χ* ^2^ = 5.523; *p*=0.019), β2-MG (*χ* ^2^ = 13.357; *p*<0.001), ApoA (*χ* ^2^ = 16.190; *p*<0.001), alkaline phosphatase (ALP) (*χ* ^2^ = 5.329; *p*=0.021), and ADA (*χ* ^2^ = 4.889; *p*=0.027). The Kaplan-Meier curves of ApoA, ADA, and ALP were shown in **Figures 1D–F**. The median PFS time of patients with low ApoA level was 57 months and was shorter than that in patients with high ApoA level (83 months). In Cox univariate analysis, Rai stage (*p*=0.011), Binet stage (*p*=0.012), LDH (*p*=0.022), β2-MG (*p*=0.011), ApoA (*p*<0.001), ALP (*p*=0.024), and ADA (*p*=0.031) were associated with PFS. Multivariate analyses showed that LDH (*p*=0.017), and ApoA (*p*<0.001) were independent predictors for PFS (shown in **Table 3**).

**Table 3:** Univariate and multivariate Cox proportion hazard model of potential prognostic parameters for progression-free survival of chronic lymphocytic leukemia patients.

Parameters		Univariate analysis			Multivariate analysis			
		P-value	HR	95%CI	P-value	HR	95%CI
**Age, years**	≥65 vs <65	0.133	1.548	0.876–2.736			
**Sex**	male vs female	0.887	0.980	0.746–1.289			
**Rai stage**	III,IV vs 0,I,II	**0.011**	**0.491**	**0.283**–**0.849**	0.236		
**Binet stage**	C vs A,B	0.012	0.498	0.290–0.858	0.124		
**LDH, U/L**	≥193.75 vs <193.75	**0.022**	**1.993**	**1.105**–**3.597**	**0.017**	**0.478**	**0.261**–**0.877**
**β2-MG, mg/L**	≥3.50 vs <3.50	0.011	2.045	1.176–3.556	0.892		
**Del13q**	positive vs negative	0.168	0.619	0.313–1.225			
**Del11q**	positive vs negative	0.936	0.969	0.445–2.110			
**Del17p**	positive vs negative	0.054	0.381	0.143–1.016			
**Tri12**	positive vs negative	0.167	0.476	0.166–1.364			
**TG, mmol/L**	≥1.10 vs <1.10	0.448	1.228	0.723–2.086			
**TC, mmol/L**	≥4.07 vs <4.07	0.452	0.815	0.479–1.387			
**HDL-C, mmol/L**	≥0.98 vs <0.98	0.093	0.626	0.363–1.081			
**LDL-C, mmol/L**	≥2.57 vs <2.57	0.976	0.992	0.584–1.685			
**sdLDL-C, mmol/L**	≥0.84 vs <0.84	0.783	0.836	0.234–2.990			
**ApoA, g/L**	≥0.98 vs <0.98	**0.000**	**0.329**	**0.185**–**0.582**	**0.000**	**3.081**	**1.659**–**5.723**
**ApoB, g/L**	≥0.86 vs <0.86	0.192	0.696	0.403–1.200			
**ApoA/ApoB**	≥1.15 vs <1.15	0.079	0.614	0.356–1.057			
**HCY, µmol/L**	≥12.40 vs <12.40	0.766	0.900	0.450–1.800			
**Lipo a, g/L**	≥0.16 vs <0.16	0.747	1.091	0.641–1.857			
**Lp-PLA2, IU/L**	≥522.50 vs <522.50	0.603	0.578	0.073–4.569			
**FFA, mmol/L**	≥0.49 vs <0.49	0.144	0.526	0.223–1.244			
**AST, U/L**	≥20.00 vs <20.00	0.482	0.827	0.486–1.406			
**ALT, U/L**	≥16.00 vs <16.00	0.251	0.730	0.427–1.249			
**SOD, U/L**	≥149.05 vs <149.05	0.093	0.584	0.311–1.095			
**GGT, U/L**	≥19.00 vs <19.00	0.229	1.388	0.814–2.369			
**ALP, U/L**	≥89.00 vs <89.00	**0.024**	**1.871**	**1.087**–**3.221**	0.764		
**GLDH, U/L**	≥2.55 vs <2.55	0.972	1.010	0.571–1.787			
**ADA, U/L**	≥13.55 vs <13.55	**0.031**	**1.876**	**1.061**–**3.317**	0.648		
**TBA, µmol/L**	≥4.50 vs <4.50	0.984	1.006	0.586–1.726			
**TBIL, µmol/L**	≥13.00 vs <13.00	0.632	0.877	0.512–1.502			
**DBIL, µmol/L**	≥2.40 vs <2.40	0.963	1.013	0.592–1.732			
**IBIL, µmol/L**	≥10.60 vs <10.60	0.701	0.900	0.525–1.542			

The bolded font means the parameters have statistical significances.

### Correlation Between ApoA Level and Treatment in CLL Patients

Of 150 patients, 90 patients received treatments during the follow-up period and data post-therapy of 59 patients were available. There was no statistical significance in ApoA level between pre-therapy and post-therapy (*p*=0.438). However, the Kaplan-Meier curves disclosed that ApoA level of patients post-therapy was still associated with OS (*χ* ^2^ = 6.732; *p*=0.009; shown in **Figure 1G**) and PFS (*χ* ^2^ = 8.822; *p*=0.003; shown in **Figure 1H**). Furthermore, we identified subgroups according to whether to use rituximab or whether to use ibrutinib. In the cohort with 21 patients who received rituximab treatment, there was no statistical significance in ApoA level between pre-therapy and post-therapy (*p*=0.955), as well as in the cohort with nine patients who received ibrutinib treatment (*p*=0.120).

### The New Risk Scoring System for CLL Patients

To optimize the risk stratification of CLL, a new risk scoring system was established based on the factors with *p ≤* 0.05 in univariate COX analysis of OS. Age, β2-MG, ApoA and ADA were included in the new risk scoring system named *ARAA-score*. Due to the different hazard ratio (HR) of the 4 factors, we allocated 2.5 points to age with an HR of 2.384 (95% CI, 1.108–5.131), β2-MG with an HR of 2.788 (95% CI, 1.137–6.833), and ApoA with an HR of 2.660 (95% CI, 1.109–6.380), and allocated 0.5 points to ADA with an HR of 0.431 (95% CI, 0.185–1.000) (shown in **Table 4**). Points were summed for each patient and a summed score range from 0 to 8 points stratified patients into 3 risk groups defined as low (0–2.5 points), intermediate (3–5 points), and high risk (5.5–8 points), with the 5-year survival rate of 93.5%, 75.6% and 30.2%, respectively (shown in **Table 5**). The Kaplan-Meier curve shows that the *ARAA-score* was associated with OS (*χ* ^2^ = 27.550; *p*<0.001; shown in **Figure 1I**) of CLL patients. We compared the *ARAA-score* with Binet stage by ROC curve. The new risk scoring system was more advanced with an area under curve (AUC) of 0.763 (95% CI, 0.672–0.854) than Binet stage with an AUC of 0.647 (95% CI, 0574–0.748) (shown in **Figure 1K**). Further study showed that there was a statistically significant association between *ARAA-score* and PFS (*χ* ^2^ = 21.794; *p*<0.001; shown in **Figure 1J**). The 5-year progression-free survival rate of low risk, intermediate risk, and high risk was 83.0%, 70.4%, and 26.0%, respectively (shown in **Table 5**). The AUC of *ARAA-score* for PFS was 0.706 (*p* < 0.001, 95% CI, 0.614–0.798), while the AUC of Binet stage for PFS was 0.636 (*p*=0.008, 95% CI, 0.542–0.729) (shown in **Figure 1L**).

**Table 4:** Allocation of risk score points of the *ARAA-score* for chronic lymphocytic leukemia patients.

Variables	Adverse factor	Points
**Age**	≥65 years	2.5
**Rai stage**	III, IV stage	2.5
**ApoA**	<0.98 mmol/L	2.5
**ADA**	≥13.55 mmol/L	0.5

**Table 5:** Risk stratification of *ARAA-score* for chronic lymphocytic leukemia patients.

Score	Risk group	5-year survival rate	5-year progression-survival rate
**0–2.5**	Low risk	93.5%	83.0%
**3–5**	Intermediate risk	75.6%	70.4%
**5.5–8**	High risk	30.2%	26.0%

### Correlation Between ApoA Level and Other Parameters

We did correlation analysis and found that increased ApoA was significantly associated with decreased β2-MG (*r* = −0.447, *p*<0.001). The ApoA level was significantly associated with Rai stage (*χ* ^2^ = 26.276; *p*<0.001) and Binet stage (*χ* ^2^ = 21.312; *p*<0.001). The low level of ApoA was related to the advanced stage. ApoA and SOD (*r*=0.290, *p*=0.002) were positive correlation, while ApoA was negatively correlated with C-reactive protein (CRP) (*r* = −0.508, *p*<0.001), ALP (*r* = −0.208, *p*=0.016), ADA (*r* = −0.278, *p*=0.001), and direct bilirubin (DBIL) (*r* = −0.194, *p*=0.024). High ApoA level was significantly correlated with tri12 positive (*χ* ^2^ = 4.270; *p*=0.039).

### The Suppressive Activity of L-4F on The Proliferation of CLL Cells

**Figure 2:**
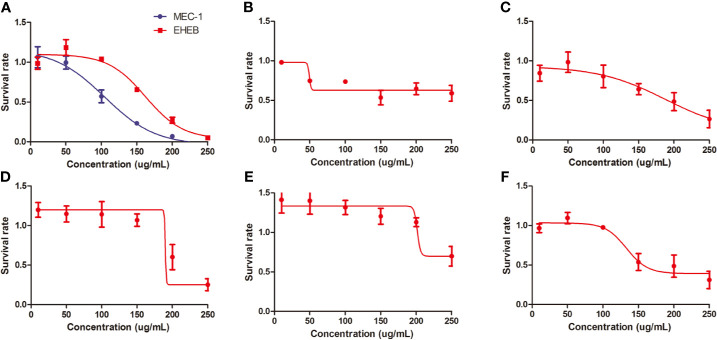
L-4F inhibits the proliferation of chronic lymphocytic leukemia (CLL) cells. **(A)** L-4F inhibits the proliferation of MEC 1 and EHEB cells at 48 h. **(B–F)** L-4F inhibits the proliferation of primary cells extracted from CLL patients marked with 1 to 5, respectively. Samples 1 and 2 were from bone marrow while Samples 3, 4, and 5 were from peripheral blood. Samples 2 and 3 were from the same CLL patient.

In vitro, we treated MEC1, EHEB cells, and primary cells with L-4F for 48 h. L-4F inhibits the proliferation of both MEC1 and EHEB cells in a dose-dependent manner (*p*<0.05), with logIC50 values of 104.6 µg/ml and 162.2 µg/ml, respectively (shown in **Figure 2A**). Samples 1 to 5 were CLL primary cells from peripheral blood or bone marrow. Of them, samples 1 and 2 were from bone marrow while others were from peripheral blood. Of note, samples 2 and 3 were from the same CLL patient. L-4F was also proven to suppress the proliferation of CLL primary cells (shown in **Figures 2B–F**).

## Discussion

Our study showed the prognostic value of ApoA in CLL and the suppressive effect of ApoA on the proliferation of CLL cells for the first time. Increased ApoA was a significant favorable prognostic factor for CLL patients. The ApoA-I mimetic peptide L-4F exhibited potent therapeutic potential in CLL. The new risk scoring system *ARAA-score* is capable of optimizing the risk stratification of CLL patients.

As the main subtype of ApoA, ApoA-I not only plays a crucial role in cholesterol transportation, anti-inflammation, and anti-oxidation, but also relates to the prognosis of some solid tumors, including breast, ovarian, lung, liver, pancreas, colon, kidney, and nasopharyngeal cancer, and so on (28). However, there was no study involving in CLL. In our study, we found that ApoA was an independent prognostic factor for OS and PFS in CLL patients. The increased ApoA was associated with the early clinical stage and tri12 positive, which are relatively favorable prognostic factors for CLL patients. Consistently, ApoA was negatively correlated with CRP, which was related to the poor OS and the development of second tumors in CLL patients (29). As shown above, our survival analyses confirm the prognostic value of ApoA in CLL.

Importantly, we unraveled the inhibitory effect of the ApoA-I mimetic peptide L-4F on CLL cell lines and primary cells. ApoA-I mimetic peptides L-4F, L-5F, and D-4F were capable of inhibiting the viability or proliferation of tumor cells in both vitro studies and vivo studies (24, 30–38). In the hematologic tumors, there was a relatively low level of HDL/ApoA-I in patients with acute lymphoblastic leukemia at diagnosis. Furthermore, patients who achieved remission after chemotherapy displayed a significant increase in ApoA-I level (11, 39, 40). L-4F can reduce the tumor burden and increase the survival of multiple myeloma by enhancing the pharmacologic value of adiponectin (13). Our study showed the therapeutic potential of ApoA in CLL.

The mechanism of ApoA-I/L-4F inhibiting the proliferation of CLL cells is unclear. It likely involves inflammatory and immune-modulatory mechanisms. Chronic inflammatory plays a pathophysiological role in CLL (41). Infections have become the major cause of morbidity and mortality among CLL patients due to immune dysfunction and cytotoxic CLL treatment (42). ApoA-I is of antiviral activity, as well as prevention for sepsis. It increases the level of an acute phase protein pertraxin3 (PTX3), which can recognize pathogen-associated molecular patterns (PAMPs) in viruses, bacteria, and fungi. Reduced ApoA-I in patients with sepsis is associated with poor survival (20). We supposed that the negative correlation between ApoA and the acute inflammatory protein CRP was related to the anti-infection activity of ApoA-I.

A possible explanation for the suppressive effect of ApoA in CLL is that ApoA-I can decrease the level of IL-6, a major mediator of inflammation, which contributes to the survival of CLL patients. Of note, plasma levels of interleukin 6 (IL-6), interleukin-8 (IL-8), interleukin-10 (IL-10), and tumor necrosis factor (TNF) in CLL patients were typically increased (20, 43). On the other hand, microenvironment plays an active role in pathogenesis, development in B-cell malignancies (44). IL-6 is expressed at high levels in the tumor microenvironment. The IL-6/JAK2/STAT3 pathway plays a crucial role in the growth and development of many cancers (41). The signal transducer and activator of transcription 3 (STAT3), a latent cytoplasmic transcription factor, shuttles to the nucleus by phosphorylation and dimerization, binds to DNA and activates STAT3-regulated genes. STAT3, phosphorylated on serine 727 in CLL cells, activates proapoptosis mechanism and induces apoptosis at high levels by eliciting the transcription of pro-survival and anti-apoptosis genes, such as Mcl-1 and Bcl-2. Both of the genes are overexpressed in CLL and the latter is a key therapeutic target of CLL (45–48).

On the other hand, a study proved that STAT3 bonded to lipoprotein lipase (LPL) promoter and activated LPL gene, which was associated with the prognosis of CLL (49, 50). Different from normal B-cells, CLL cells produce chemical energy by utilizing FFAs, and there is an increased level of FFAs and TG degradation products. LPL, catalyzing the hydrolysis of TG into FFA, was found aberrant expression in CLL cells, which was similar to fat and muscle cells on signatures. It mediates the uptake of cholesterol, therefore reduces the level of cholesterol, HDL, VLDL, and TG (14, 17, 50–53). For this reason, the inhibition of STAT3 pathway is likely to inhibit the utilization of FFA in CLL cells.

**Figure 3:**
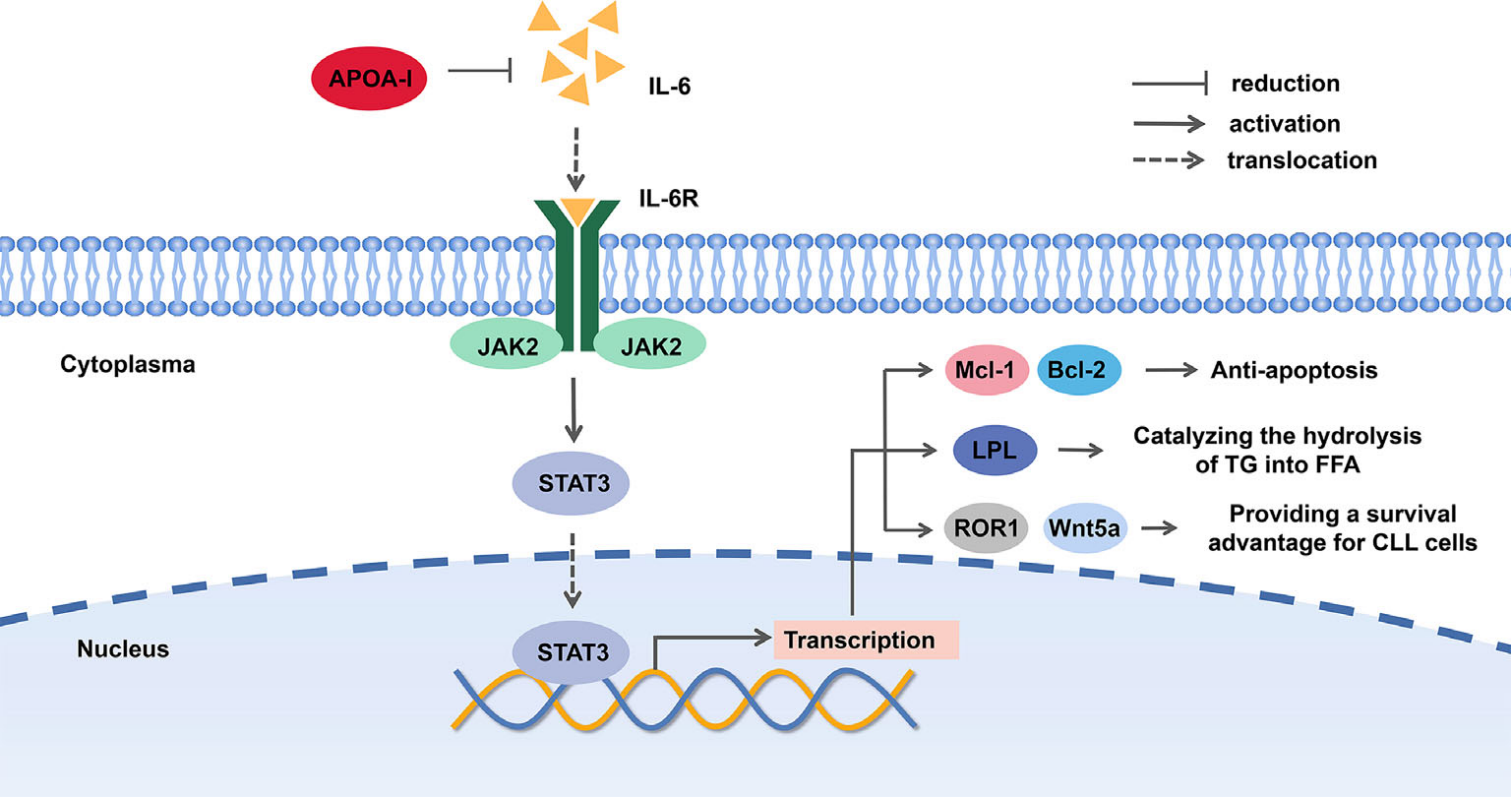
The potential mechanism of Apolipoprotein A-I (ApoA-I) inhibition chronic lymphocytic leukemia (CLL) cells.

Furthermore, STAT3 can induce the expression of receptor tyrosine kinase-like orphan receptor-1 (ROR1) and Wnt5a, a member of the wingless and integration site growth factor (WNT) family in CLL cells. The aberrant Wnt/β-catenin signaling pathway promotes cancer stem cell renewal, cell proliferation, and differentiation. STAT3-induced Wnt5a was disclosed to provide CLL cells with a microenvironment-independent survival advantage (54, 55). Hence, we proposed that ApoA-I inhibited JAK2/STAT3 pathway by decreasing IL-6 level to suppress CLL cells (shown in **Figure 3**).

Besides, the inhibition of JAK2/STAT3 pathway may enhance the effect of ibrutinib (47). Inhibition of fatty acid oxidation reduced ibrutinib resistance (56). Thus, ApoA-I/L-4F is a potential therapy for CLL patients and may improve ibrutinib efficacy. However, the findings of the current study that there was no statistical difference in ApoA between pre-therapy and post-therapy do not support the previous research. The suppressive effect and mechanism of ApoA-I in CLL cells needs further study.

HDL and lipid-free ApoA-I contributes to the reduction of lipid raft abundance by reverse cholesterol transport. CD19 is a B cell-specific antigen expressed on CLL cells and serving as a co-stimulatory molecule for B-cell receptor (BCR) signaling (57). It prolongs BCR signaling in lipid rafts, which are detergent-resistant, sphingolipid, and cholesterol-rich membrane fractions (58). Dysregulation of lipid rafts plays a key role in the pathogenesis of inflammation, hematopoietic disease, and cancer. Lipid rafts provide a novel therapeutic target for the disease (59). It may be the potential mechanism of ApoA-I and its mimetics to inhibit the co-ligation of CD19 and BCR.

Our study showed the correlation between SOD and ApoA. SOD is a superoxide scavenger and may be important to normal vascular function and cardiovascular health. This finding is consistent with that of Mahaney, M. C. who demonstrated the pleiotropy between SOD and ApoA-I (60). On the other hand, D-4F was reported to inhibit the proliferation of ovarian tumor cell line ID8 cell and ovarian tumor development by inducing the expression and activity of mitochondrial manganese (MnSOD), which is the primary antioxidant enzyme that protects cells from reactive oxygen species (ROS) inducing damage to mitochondrial element injury (35, 61). HDL-associated ApoA-I attenuates mitochondrial injury by the suppression of ROS formation and induction of autophagy, which may be another mechanism by which ApoA-I inhibits the proliferation of CLL cells.

Risk stratification in CLL is vital for treatment decisions. Rai stage and Binet stage are the most widely used staging system in clinical practice. There are increasing prognostic models that have been developed to improve the prognosis of CLL (6, 62). The *ARAA-score* we established had prognostic value for both OS and PFS of CLL patients. ADA was associated with OS of CLL patients. A prior study revealed that serum ADA activity was found higher in CLL patients than in control. It was reported to be correlated to β2-MG, LDH, white blood cell (WBC), and erythrocyte sedimentation rate (ESR) (63, 64). Fludarabine, against lymphoid malignancies, particularly CLL and low-grade non-Hodgkin lymphoma (NHL), is with an advantage of resistance to ADA deamination (65). Thus, ADA was also included in the *ARAA-score*. The parameters included in the new risk scoring system are both available easily and cheap. ROC curves showed that the *ARAA-score* is more accurate in risk stratification than Binet stage. However, our study was a single-center study, and no cytogenetic aberration was included.

In conclusion, the study shows that ApoA is an independent prognostic factor for both OS and PFS in CLL. The ApoA-I mimetic L-4F inhibits the proliferation of CLL cells *in vitro*. Besides, age, Rai stage, ApoA, and ADA are included to develop a new risk scoring system named *ARAA-score*. It will optimize the risk stratification of CLL patients. However, the use of ApoA-I in CLL therapy and *ARAA-score* needs further study.

## Data Availability Statement

The raw data supporting the conclusions of this article will be made available by the authors, without undue reservation.

## Ethics Statement

The studies involving human participants were reviewed and approved by Medical Ethical Committee of Shandong Provincial Hospital affiliated to Shandong University. Written informed consent for participation was not required for this study in accordance with the national legislation and the institutional requirements.

## Author Contributions

XW and YZ designed the study. XY, XS, HZ, ZY, XZ, and ML collected the clinical data. XY and XH performed the cell proliferation assays. XY analyzed the data and wrote the paper. YZ and XW revised the paper. All authors contributed to the article and approved the submitted version.

## Funding

This study was funded by National Natural Science Foundation (82000195, 82070203, 81770210, 81473486, and 81270598); Key Research and Development Program of Shandong Province (2018CXGC1213); Technology Development Projects of Shandong Province (2017GSF18189); Translational Research Grant of NCRCH (2021WWB02, 2020ZKMB01); Shandong Provincial Natural Science Foundation (ZR2020QH094); Taishan Scholars Program of Shandong Province; Shandong Provincial Engineering Research Center of Lymphoma; Academic Promotion Programme of Shandong First Medical University (2019QL018 ; 2020RC007); Technology Development Project of Jinan City (202019182); Shandong Provincial Hospital Youth Talent Plan; Shandong Provincial Hospital Research Incubation Fund.

## Conflict of Interest

The authors declare that the research was conducted in the absence of any commercial or financial relationships that could be construed as a potential conflict of interest.
